# Classroom behavioral indicators predict academic performance in large-class clinical instruction: a cohort study using real-time behavioral data

**DOI:** 10.1186/s12909-026-09340-2

**Published:** 2026-04-29

**Authors:** Shiying Sun, Shenglin Xu, Xue Wang

**Affiliations:** Department of Gynecology and Obstetrics, The Second Affiliated Hospital of Anhui Medical University, 678 Furong Road, Hefei, 230601 P. R. China

**Keywords:** Medical education, Student engagement, Learning analytics, Assessment format, Quiz completion

## Abstract

**Introduction:**

Understanding how in-class behaviors relate to academic performance is critical for improving instructional effectiveness in medical education. This study examined behavioral indicators collected via a digital teaching platform to identify meaningful predictors of academic success in an undergraduate obstetrics and gynecology course.

**Methods:**

A total of 1,178 fourth-year medical students from the 2020 and 2021 entry cohorts were included. Individual-level analyses were conducted using data from 1,048 students with valid behavioral and quiz-response records. Four behavioral indicators were tracked through Rain Classroom: attendance rate, quiz completion rate, answer accuracy, and student engagement. Univariate and multivariate linear regression analyses were used to assess associations between behavioral indicators (quiz completion rate and student engagement score) and answer accuracy at the individual student level. Assessment format (examination-based vs. non-examination-based) was additionally included as a contextual variable in the analyses. Descriptive comparisons at the class level were also performed.

**Results:**

At the individual student level, quiz completion rate (*r* = 0.3497, *df* = 1046, *p* < 0.0001), student engagement score (*r* = 0.5095, *df* = 1046, *p* < 0.0001), and assessment format (*r* = 0.2881, *df* = 1046, *p* < 0.0001) were all significantly associated with answer accuracy. In multivariate analysis, all three variables remained independently associated with answer accuracy, including student engagement score (*B* = 0.0297, *p* < 0.0001), quiz completion rate (*B* = 0.1020, *p* = 0.0003), and assessment format (*B* = 3.473, *p* = 0.0264). Student engagement score showed the strongest association with answer accuracy among the behavioral indicators.

**Conclusion:**

Real-time behavioral analytics can provide actionable insights into learning dynamics and highlight the instructional importance of fostering active engagement. In addition, quiz completion and assessment format were also associated with student performance, suggesting that both in-class behavioral participation and assessment context contribute to learning outcomes. These findings support the alignment of engagement-focused strategies and assessment designs to improve learning outcomes in high-enrollment clinical education contexts.

## Introduction

Obstetrics and Gynecology is a foundational subject in undergraduate medical education, delivering essential knowledge on female reproductive physiology, pregnancy, childbirth, and gynecologic conditions [1, 2]. As a compulsory theoretical course, it plays a pivotal role in preparing students for clinical practice and national medical licensing examinations [3]. However, due to the complexity of the content and the typically large class sizes in preclinical teaching, students often encounter difficulties engaging with the material and maintaining motivation during traditional didactic lectures. Student engagement has been conceptualized as a multidimensional construct encompassing behavioral, emotional, and cognitive components [4, 5]. Within this framework, observable indicators such as attendance or task completion primarily reflect behavioral participation, whereas cognitive engagement involves sustained attention, strategic effort, and active knowledge construction. Importantly, these dimensions are related but not interchangeable; visible compliance does not necessarily indicate deeper cognitive processing. In digitally mediated classrooms, individual-level behavioral log data offer an opportunity to differentiate between procedural participation and interactive engagement, thereby enabling more precise examination of how specific forms of engagement relate to academic performance [6]. Passive learning environments, characterized by limited interactivity and one-way information transmission, have been associated with weaker conceptual understanding and reduced long-term retention [7]. From a cognitive learning perspective, deeper conceptual understanding depends on active processing, integration, and elaboration of information. When instructional settings limit opportunities for interaction and generative engagement, learners may rely primarily on surface-level processing, which constrains durable knowledge construction [4, 5]. These challenges underscore the need to explore more effective methods of delivering theoretical instruction in medical curricula.

In recent years, the integration of digital platforms into medical education has gained momentum, particularly those capable of supporting real-time feedback, interactive engagement, and individualized monitoring [8]. Among these, Rain Classroom, a WeChat-integrated mini program jointly developed by Tsinghua University and the Xuetang Online Company, has been widely adopted across Chinese universities [9]. Designed to integrate seamlessly with Microsoft PowerPoint and mobile devices, the Rain Classroom platform allows instructors to deliver quizzes, share learning resources, collect feedback, and monitor classroom dynamics during live sessions [10]. These features are particularly well suited for large-group theoretical teaching, where formative assessment and engagement analytics can help bridge the gap between instructional delivery and student learning.

While the technical functionalities of platforms like Rain Classroom are increasingly acknowledged, many evaluations of teaching effectiveness, particularly in large-group medical lectures, continue to rely on static indicators such as attendance and task completion, which may not fully capture students’ cognitive or interactive engagement [11]. In contrast, behavior-based metrics, such as the frequency of real-time interactions, slide annotations, and participation in in-class activities, may offer more meaningful insights into learning dynamics [12, 13]. However, quantitative evidence linking individual-level, real-time classroom behavioral data to objective academic performance remains limited, particularly in large-group clinical teaching contexts.

Although Rain Classroom has been widely implemented in Chinese medical education, existing studies have primarily examined students’ subjective experiences, such as satisfaction, perceived engagement, or general course outcomes, most often through self-reported questionnaires [8]. Empirical investigations that quantitatively link real-time classroom behaviors with measurable academic outcomes remain limited [14]. This lack of evidence is particularly notable in clinical theory courses, where both content mastery and active student engagement are essential. In this context, performance on in-class quizzes serves as a quantifiable proxy for students’ theoretical understanding, especially in disciplines such as Obstetrics and Gynecology.

To address this gap, the present study examines the association between individual-level classroom behavioral indicators and in-class quiz performance in a fourth-year Obstetrics and Gynecology course delivered via Rain Classroom at Anhui Medical University. Specifically, we analyzed key behavioral indicators, including quiz completion rate and student engagement score, and answer accuracy. In addition, assessment format (examination-based vs. non-examination-based) was included as a contextual variable to examine its association with student performance. Given prior literature indicating that assessment structure influences student motivation and effort allocation, this comparison was conducted to examine whether differences in evaluative context were associated with distinct behavioral engagement profiles. By identifying which behavioral indicators best predict theoretical performance, this study aims to inform evidence-based strategies for optimizing digital instruction and enhancing engagement in clinical medical education.

## Methods

### Study design and setting

This retrospective observational study was conducted at Anhui Medical University and focused on the delivery of the Obstetrics and Gynecology course to fourth-year undergraduate students. The course adopted a blended instructional model, combining traditional lectures with interactive elements through Rain Classroom, a digital platform jointly developed by Tsinghua University and Xuetang Online [8]. Although the cohorts are designated as the 2020 and 2021 entry classes, the instructional modules analyzed in this study were delivered during the autumn semesters of 2023 and 2024, respectively. Data were collected from three thematic modules: Prenatal Examination and Pregnancy Care, Genetic Counseling and Prenatal Screening, and Fetal Intervention. Each module consisted of a 120-minute session delivered by the same instructor using a standardized syllabus based on the 9th edition of *Obstetrics and Gynecology* (People’s Medical Publishing House). All student groups received identical instructional content and completed the same objective in-class quiz administered via Rain Classroom during the session. The quiz consisted of an identical set of questions across programs, was administered under comparable classroom conditions, and was scored using a unified automated grading system.

In addition to examining behavioral predictors of academic performance, we conducted a supplementary comparison between examination-based and non-examination-based course formats. This comparison was performed at the class level and represents a between-group observational analysis rather than a within-subject design. Students in examination-based and non-examination-based formats belonged to different teaching groups.

### Participants

A total of 1,178 fourth-year undergraduate students from two academic cohorts (2020 and 2021) were included in this study. Students were enrolled in one of four programs: Clinical Medicine (divided into Class A and Class B), “5 + 3” Integrated Training, Anesthesiology, and Rehabilitation Medicine. The 2020 cohort included 581 students, and the 2021 cohort included 597. All students received identical course content under standardized teaching conditions.

All 1,178 students were included in the descriptive summaries and class-level analyses (*n* = 10 classes). For individual-level univariate and multivariable analyses, a subsample of 1,048 students with complete records for the relevant behavioral variables and in-class quiz responses were included. Students were excluded from these individual-level analyses if they did not complete the platform sign-in process successfully or generated no analyzable interactive or quiz-response data during the session.

### Data collection and indicators

Behavioral data were obtained from Rain Classroom, which automatically logs student interactions during class [15]. During each session, instructors delivered slides through Microsoft PowerPoint integrated with Rain Classroom and embedded real-time quizzes and interactive prompts within the presentation. Students accessed the session via their mobile devices and responded to in-class questions, participated in live polls, and interacted with slide content in real time. The platform automatically recorded these interaction events and generated session-level behavioral logs for subsequent analysis.

The following four behavioral indicators were analyzed: attendance rate, quiz completion rate, answer accuracy, and student engagement score [12]. Attendance rate was determined by completion of the platform’s sign-in process. Quiz completion rate represented the proportion of in-class quizzes attempted. Answer accuracy referred to the percentage of correct responses on in-class quizzes. In this study, academic performance was operationalized as this measure, reflecting students’ immediate understanding of the instructional content. Student engagement score was a composite index generated by the system, incorporating in-class behaviors such as slide marking, responding to questions, commenting, and participating in live polls. In addition, assessment format (examination-based vs. non-examination-based) was included as a contextual variable and coded as a binary variable for regression analyses. The score reflects the cumulative frequency of interactive behaviors automatically recorded during each teaching session. Engagement data were extracted as session-level behavioral logs and analyzed as aggregated class-level metrics. For class-level analyses, behavioral indicators were summarized as mean values within each teaching class. For univariate and multivariable analyses, quiz completion rate, answer accuracy, and student engagement score were analyzed at the individual student level. Although the exact weighting algorithm used by the platform is proprietary and not publicly disclosed, the index is derived from observable interaction events captured by the system without manual adjustment.

Students were not excluded using a generic “incomplete data” rule. Instead, for individual-level analyses, we included only students with analyzable records for the variables of interest, and excluded those without a successful sign-in record or without any recorded interactive or quiz-response data in Rain Classroom. All exported data were reviewed for internal consistency and completeness before analysis.

### Ethical considerations

This study involved the secondary analysis of anonymized classroom data collected during regular instruction. No personally identifiable information was accessed or retained. As the study involved retrospective analysis of de-identified routine teaching data and did not include any intervention, formal ethical review and individual informed consent were not required under institutional policy.

### Statistical analysis

**Fig. 1:**
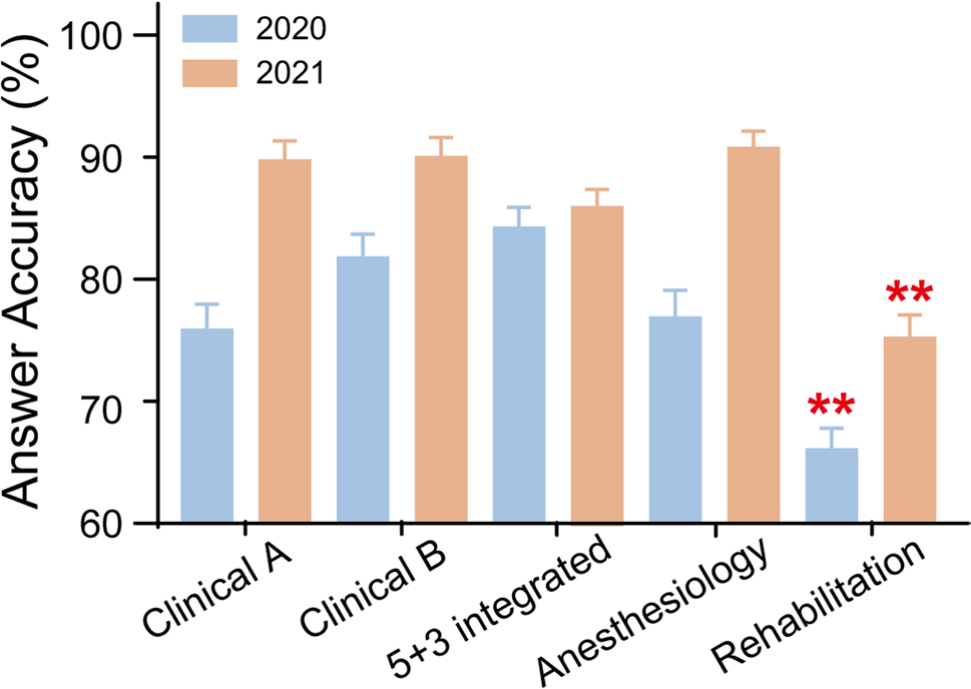
Comparison of answer accuracy across academic classes and cohorts. Bar chart showing the mean quiz answer accuracy (%) of students from ten academic classes, grouped by cohort (2020 or 2021) and academic program (Clinical A, Clinical B, “5 + 3” integrated training, Anaesthesiology, and Rehabilitation). Students in the Rehabilitation classes showed consistently lower answer accuracy, while Clinical and Anaesthesiology classes performed better. Statistical significance was assessed using one-way ANOVA with Tukey’s post hoc test; asterisks denote classes with significantly different accuracy levels (*p* < 0.01)

Statistical analyses were conducted using GraphPad Prism 9.0 (GraphPad Software Inc., USA). Descriptive statistics for class-level summaries are presented as means and standard deviations, where applicable. One-way ANOVA with Tukey’s post hoc tests was used to compare answer accuracy across groups. Figure 1 was based on class-level data, with each data point representing the mean value of a teaching class (*n* = 10). Univariate associations between behavioral variables and answer accuracy were examined at the individual student level using simple linear regression. For these analyses, data from all classes were pooled, and each data point represented one student (*n* = 1,048). Multivariable linear regression was then performed at the individual student level to identify independent predictors of answer accuracy. Multicollinearity was assessed using variance inflation factors; *p* < 0.05 was considered statistically significant. The association between assessment format and answer accuracy was evaluated using point-biserial correlation, with assessment format coded as a binary variable.

## Results

### Student characteristics and overall performance

A total of 1,178 fourth-year undergraduate medical students were included in the overall study sample and class-level descriptive analyses, comprising 581 students from the 2020 cohort and 597 from the 2021 cohort. Each cohort was divided into five teaching classes according to academic program: Clinical Class A, Clinical Class B, “5 + 3” integrated training, Anesthesiology, and Rehabilitation. At the class level, four indicators were summarized: attendance rate, quiz completion rate, answer accuracy, and engagement score, as summarized in Table 1. Students from the 2021 cohort showed slightly higher mean values across all four indicators compared to those from the 2020 cohort. However, these differences were not statistically significant at the class level.

**Table 1:** Class-level summary of behavioral indicators by cohort and teaching class

Cohort	Class	Students	Attendance (%)	Quiz Completion (%)	Accuracy (%)	Class-level Engagement Score
2020	Clinical A	156	97	87	76	554
2020	Clinical B	155	92	77	82	477
2020	5 + 3 integrated	90	95	93	84.4	345
2020	Anaesthesiology	127	77	76	77	321
2020	Rehabilitation	53	96	86	66	173
2021	Clinical A	162	95	78	90	581
2021	Clinical B	166	92	87	90	677
2021	5 + 3 integrated	90	98	96	86	383
2021	Anaesthesiology	121	96	97	91	553
2021	Rehabilitation	58	98	87	75	224

Values are presented as class-level mean percentages for attendance, quiz completion, and answer accuracy, and mean engagement scores for each teaching class

### Class-level differences in answer accuracy

To further explore differences in student performance, answer accuracy was compared across the ten teaching classes in the two cohorts (Fig. 1). Substantial inter-class variation was observed. In both cohorts, students from the Rehabilitation classes consistently demonstrated the lowest answer accuracy (66% in 2020 and 75% in 2021), while those in the Anesthesiology and Clinical Class A groups achieved higher levels, approaching 90%. One-way ANOVA confirmed that the differences in answer accuracy across classes were statistically significant (*F* (9, 1168) = 12.45, *p* < 0.001). Post-hoc Tukey HSD tests further indicated that the Rehabilitation class differed significantly from all other groups (*p* < 0.01), and no significant differences were found among the remaining classes (*p* > 0.05). These results suggest class-level heterogeneity in answer accuracy across teaching groups. Notably, the persistent underperformance of the Rehabilitation group highlights the need for targeted pedagogical support.

### Individual-level univariate analysis of behavioral indicators

**Fig. 2:**
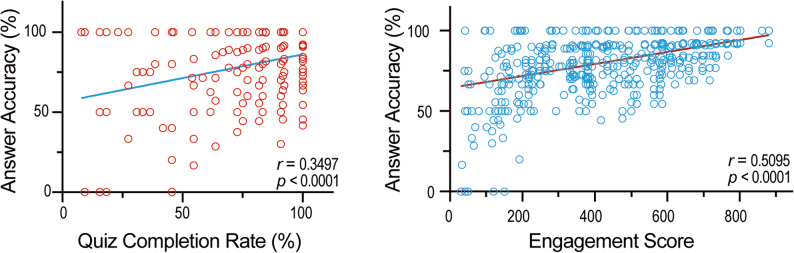
Correlation between behavioral indicators and answer accuracy. Scatter plots showing the relationship between quiz completion rate (left) and student engagement score (right) and in-class quiz answer accuracy at the individual student level (*n* = 1,048; *df* = 1,046 for each analysis). Each point represents one student. Data from all classes were pooled and analyzed at the individual student level. Linear regression lines are shown. Correlation coefficients (*r*), degrees of freedom (*df*), and corresponding *p*-values are indicated within each panel

To identify which classroom behaviors were most strongly associated with in-class quiz performance, individual-level univariate analyses were conducted using data pooled across all classes for students with analyzable behavioral and quiz-response records (*n* = 1,048). As shown in Fig. 2, both quiz completion rate (*r* = 0.3497, *df* = 1046, *p* < 0.0001) and student engagement score (*r* = 0.5095, *df* = 1046, *p* < 0.0001) showed significant positive correlations with answer accuracy. These findings indicate that, at the individual student level, both completion of in-class quizzes and active engagement during class were associated with better answer accuracy. Compared with quiz completion rate, student engagement showed a stronger association with answer accuracy, suggesting that more active and interactive forms of participation may better reflect effective learning.

### Individual-level association between assessment format and performance

**Fig. 3:**
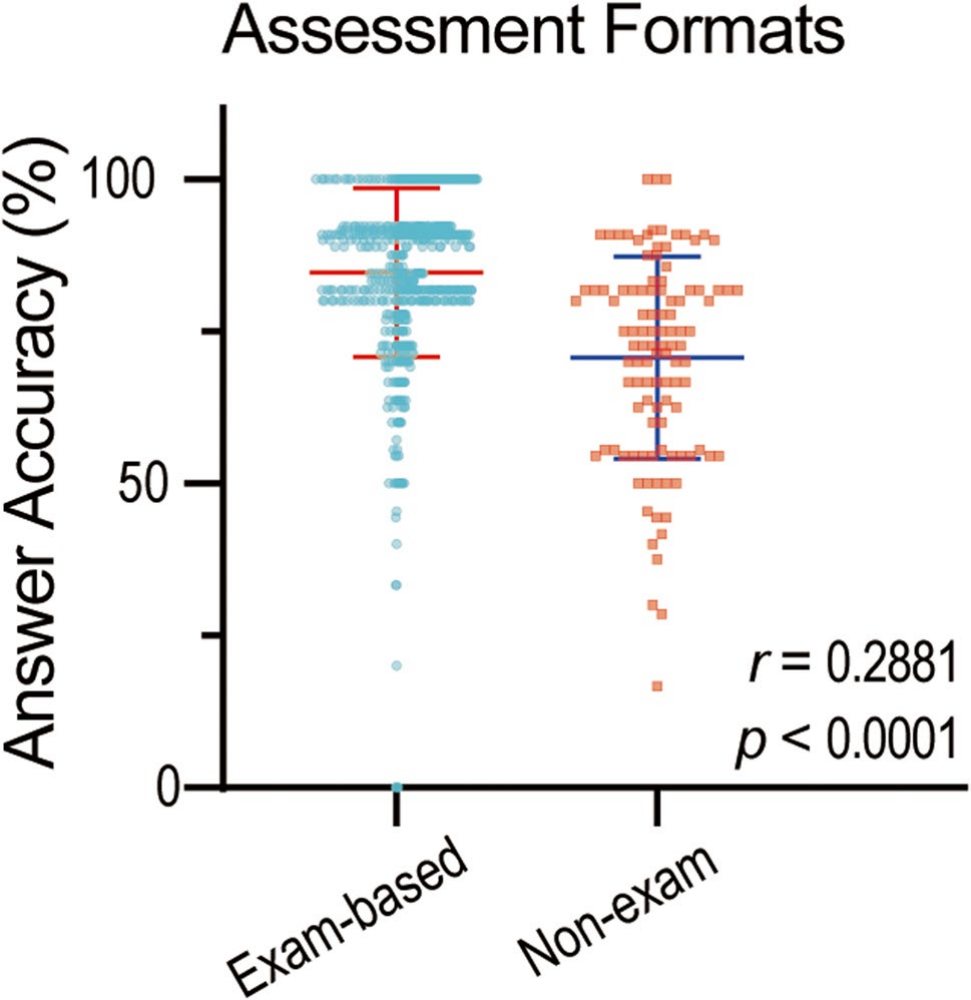
Relationship between assessment format and answer accuracy. Scatter plot of student answer accuracy (%) by assessment format. Each point represents one student (*n* = 1,048; *df* = 1,046). Assessment format was coded as a binary variable, and the association with answer accuracy was evaluated using point-biserial correlation (*r* = 0.2881, *p* < 0.0001)

To further examine whether instructional context was also associated with in-class quiz performance, assessment format was analyzed at the individual student level as a binary variable. As shown in Fig. 3, a significant positive association was observed between examination-based format and answer accuracy (*r* = 0.2881, *df* = 1046, *p* < 0.0001). This finding suggests that assessment format may influence student performance, with examination-based courses associated with higher answer accuracy than non-examination-based courses.

### Multivariate analysis of predictors of accuracy

**Fig. 4:**
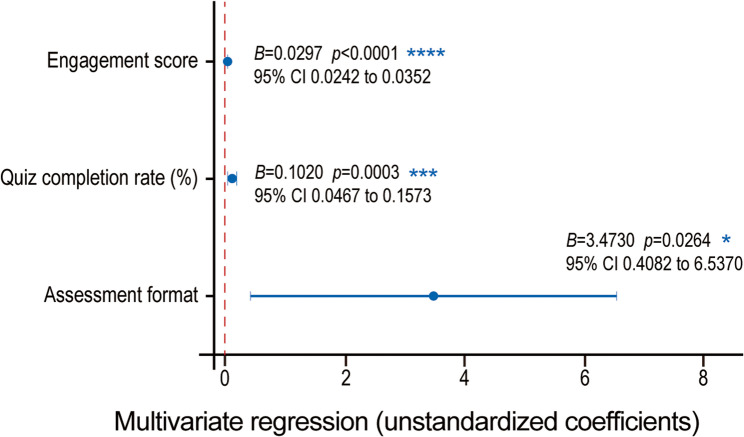
Multivariate regression of behavioral indicators on answer accuracy. Forest plot showing unstandardized regression coefficients (*B*) from the multivariable linear regression model predicting answer accuracy. Points represent unstandardized coefficients, and horizontal bars indicate 95% confidence intervals. Corresponding p-values are shown alongside each predictor. (*p < 0.05, ***p < 0.001, ****p < 0.0001)

To further assess the independent contributions of behavioral and contextual variables, a multivariate linear regression model was constructed at the individual student level using student engagement score, quiz completion rate, and assessment format as predictors, with answer accuracy as the outcome. The model was statistically significant (*F* (3, 1044) = 128.6, *p* < 0.0001), explaining approximately 27% of the variance in answer accuracy (*R*² = 0.2698). As shown in Table 2; Fig. 4, all three predictors were significantly associated with answer accuracy, including student engagement score (*B* = 0.0297, *p* < 0.0001), quiz completion rate (*B* = 0.1020, *p* = 0.0003), and assessment format (*B* = 3.473, *p* = 0.0264). All variance inflation factor values were below 3, indicating no notable multicollinearity. These findings suggest that both behavioral indicators and assessment format independently contributed to variation in answer accuracy, with student engagement remaining the strongest and most consistent predictor.

**Table 2:** Multivariate linear regression analysis of individual-level answer accuracy

Predictor	*B*	*p*-value	VIF
Engagement score	0.0297	< 0.0001	2.127
Quiz completion rate (%)	0.1020	0.0003	1.577
Assessment format	3.473	0.0264	1.486

Model statistics: *F* (3, 1044) = 128.6, *p* < 0.0001; *R²* = 0.2698

## Discussion

In contemporary medical education, identifying meaningful indicators of student engagement is increasingly essential for evaluating instructional effectiveness and guiding curriculum development [4]. In this study, we examined the relationship between individual-level classroom behavioral indicators collected via Rain Classroom and in-class quiz performance in a large-class clinical teaching context. Through automated data collection coupled with outcome-based assessment, we systematically examined the predictive value of key behavioral indicators. Notably, student engagement, defined by real-time participation, slide interactions, and feedback responsiveness, emerged as the most consistent and robust predictor of answer accuracy [5, 6]. These findings underscore the importance of fostering active classroom involvement, rather than relying solely on passive compliance, to promote effective learning outcomes. Quiz completion is commonly used as a behavioral proxy for learning engagement [16]. Our findings suggest that it was positively associated with answer accuracy at the individual student level. Although quiz completion may partly reflect procedural participation, in the present real-time classroom context it may also indicate task responsiveness and active involvement in instructional activities. This pattern is consistent with prior learning-analytics evidence suggesting that interactive behavioral log data better capture meaningful learning processes than static participation counts [6, 12]. Taken together, these findings indicate that individual-level platform-recorded behaviors can provide useful information about variation in students’ in-class learning performance.

In contrast, student engagement score, operationalized through real-time participation, slide interactions, and feedback responsiveness, demonstrated the strongest and most consistent relationship with answer accuracy [5]. In this study, engagement was operationalized as system-recorded interactive behavior rather than self-reported perception, thereby focusing on observable participation patterns within the classroom environment. Notably, these indicators were enabled by Rain Classroom, a digital teaching platform that facilitates the comprehensive and simultaneous collection of both behavioral and performance data from every student during class sessions. Unlike conventional oral questioning, which reveals understanding at the individual level and in limited samples, Rain Classroom allowed instructors to access immediate accuracy distributions across all students, track both individual and aggregate responses, and identify knowledge gaps in real time [17]. These capabilities not only enhanced the ecological validity of our engagement measures but also offer scalable approaches to formative assessment in large-enrollment instructional settings. Our finding that engagement showed a stronger association with accuracy than quiz completion rate and remained the most influential predictor in the multivariate model aligns with engagement frameworks in health professions education, which emphasize that observable interactive participation can more directly reflect learning-relevant behaviors than mere presence [4, 5].

In addition to individual behavioral indicators, the assessment format itself appeared to influence academic outcomes [18]. At the individual student level, examination-based course format was positively associated with answer accuracy in both the univariate and multivariate analyses. This pattern suggests that the evaluative context may shape how students allocate effort and respond to classroom learning tasks. These findings are consistent with existing literature suggesting that summative assessments may promote sustained attention and cognitive effort by reinforcing accountability and clarifying performance expectations [19]. In contrast, when formal evaluations are absent, students may perceive a lower sense of academic stakes, leading to reduced engagement even when course content and delivery are comparable. These results highlight the need for intentional alignment between assessment strategies and instructional goals, particularly in technology-enhanced classrooms where disengagement may not be immediately visible to instructors. More explicitly, our findings align with the broader view that assessment design shapes how learners allocate effort and engage with learning tasks, which has been discussed as a key lever for instructional improvement [18, 19]. However, because this comparison was observational and assessment format was determined by the existing course context rather than random allocation, causal inference cannot be established. The observed differences should therefore be interpreted as associations related to assessment context rather than direct effects of examination format.

Student engagement score, quiz completion rate, and assessment format were all significantly associated with answer accuracy in the multivariate model. This finding is consistent with prior research highlighting that active participation and cognitive engagement are more strongly linked to meaningful learning outcomes than superficial forms of compliance, such as merely attending class or submitting assignments [5, 20]. In the present study, both engagement and completion retained independent associations with answer accuracy, suggesting that these behavioral indicators captured complementary aspects of students’ in-class learning processes. The predictive strength of engagement likely stems from its integrative nature. It reflects not only attentiveness but also real-time responsiveness and voluntary interaction with instructional content [21]. These elements represent more authentic indicators of learning than passive behaviors. At the same time, the independent contribution of quiz completion rate suggests that timely participation in in-class tasks may support performance by increasing opportunities for retrieval, response generation, and ongoing knowledge reinforcement. Assessment format also retained an independent association with answer accuracy, indicating that contextual features of course design may shape learning outcomes beyond students’ immediate classroom behaviors. Accordingly, instructors aiming to improve student outcomes may benefit more from cultivating interactive and participatory classroom environments and integrating structured in-class tasks with clear evaluative expectations than from enforcing participation through administrative measures alone. This interpretation is consistent with studies showing that active, feedback-rich participation supports learning via mechanisms such as retrieval practice and timely error correction, which are less likely to occur when students engage only procedurally [20, 21].

Although this study identified student engagement as a key behavioral predictor of in-class performance, the observed variability across student groups underscores the importance of considering individual and contextual differences [5]. All participants received uniform instruction, materials, and assessments within a centralized curriculum, yet their engagement levels and accuracy outcomes were not uniform. Such discrepancies may arise from differences in learning preferences, perceived relevance of the course content, or classroom dynamics [20]. The persistently lower performance among Rehabilitation Medicine students may reflect differences in career orientation or the perceived applicability of obstetrics content, despite comparable admission standards and instructional exposure [13]. At the same time, alternative explanations should also be considered. Although all student groups completed the same objective in-class quiz under comparable classroom conditions and responses were scored using a unified automated grading system, differences in broader assessment context or program-specific learning orientation may still have contributed to the observed variation. Notably, students across different academic programs had comparable baseline academic backgrounds, including similar admission criteria and shared foundational training, suggesting that prior academic ability was unlikely to be the primary driver of the observed differences. Instead, it is plausible that discipline-specific academic orientation or professional identity development may influence how students interpret and respond to course demands, even when exposed to the same instructional strategies [22, 23]. Importantly, all student groups received identical instructional content and completed the same objective in-class quiz under comparable classroom conditions, with responses scored using a unified automated grading system. Therefore, differences in question difficulty, grading procedures, or instructional exposure are unlikely to account for the observed variation.

It should also be noted that while Rain Classroom enables efficient and large-scale behavioral data collection, it may not fully capture students’ cognitive depth or self-directed learning that occurs outside formal classroom sessions. In addition, because the engagement score is generated through a proprietary algorithm, the precise weighting of individual interaction behaviors cannot be independently verified. Although the score is based on automatically recorded observable events, this limitation may affect strict methodological replicability across different digital platforms. Future studies may benefit from a mixed-methods approach, combining digital analytics with qualitative inquiry to better understand how individual variation interacts with technology-enhanced learning environments [24].

## Conclusion

This study examined the relationship between individual-level classroom behavioral indicators and answer accuracy in an undergraduate obstetrics and gynecology course. Among the variables assessed, student engagement score demonstrated the strongest association with answer accuracy, while quiz completion rate and assessment format were also significantly associated with answer accuracy in both univariate and multivariate analyses, emphasizing the pedagogical importance of fostering active participation during instruction. By leveraging in-class behavioral data collected via Rain Classroom, instructors were able to monitor learning dynamics at scale and respond to performance trends more effectively. These findings suggest that integrating real-time engagement analytics into classroom instruction can support more responsive, data-informed teaching decisions, particularly in high-enrollment clinical courses where individualized instructional support may be constrained. At the same time, the observed role of assessment format highlights the importance of aligning evaluative design with instructional objectives to promote effective learning behaviors. These insights may help inform future instructional strategies aimed at fostering engagement in large-group clinical teaching.

## Data Availability

The dataset contains de-identified educational behavioral data derived from institutional teaching records. Because the data originate from student educational records, they are not publicly posted under current institutional data-protection regulations. A de-identified individual-level analytic dataset, together with the relevant variable definitions needed to reproduce the reported analyses, is available from the corresponding author upon reasonable request for academic research purposes, subject to institutional approval and a data-use agreement.
